# Irreducible posterior fracture-dislocation of the hip associated with an ipsilateral femoral shaft fracture: A case report and review of the literature

**DOI:** 10.1016/j.ijscr.2024.110365

**Published:** 2024-10-02

**Authors:** Imad Jadib, Houssam Eddine Rachidi, Soufiane Abdennaji, Abdeljebbar Messoudi, Mohamed Rafai

**Affiliations:** Department of Orthopedics and Trauma-Surgery (P32), Ibn Rochd University Hospital Center, Faculty of Medicine and Pharmacy Hassan-II, Casablanca, Morocco

**Keywords:** Irreducible hip fracture-dislocation, Ipsilateral fracture shaft of femur, Open reduction, Intramedullary nailing, Case report

## Abstract

**Introduction:**

Ipsilateral fractures of the shaft of the femur combined with hip dislocations are extremely rare injuries, presenting a difficult diagnostic and therapeutic challenge. Diagnosis of hip dislocation is often delayed, due to the focus on femoral fracture.

**Case presentation:**

This article presents a case never described before of a 19-year-old patient who sustained this unusual combination of injuries as a result of a road traffic accident. The patient had an irreducible posterior hip fracture-dislocation associated with an ipsilateral femoral shaft fracture. Closed reduction attempts were unsuccessful, leading to the requirement for open reduction, via the posterolateral approach of the hip, which revealed that the femoral head buttonholed through the capsule. We proceeded to the reduction of the left hip with the osteosynthesis of the posterior wall acetabular fragment, and then the femur shaft fracture was fixed using an intramedullary nail. After 19 months postoperative follow-up, the patient had full range motion of the affected hip without any pain.

**Discussion:**

Closed reduction techniques, including various external devices, have been explored, with some success in specific cases. However, open reduction remains a crucial option, especially in irreducible dislocations. Complications, such as avascular necrosis of the femoral head and neurovascular injury, illustrate the importance of accurate diagnosis and appropriate treatment.

**Conclusions:**

In conclusion, ipsilateral femoral shaft fractures combined with hip dislocations represent a rare and challenging orthopedic emergency. Timely diagnosis, careful assessment, and consideration of both closed and open reduction techniques are essential in managing these complex injuries.

## Highlights

• Ipsilateral fractures of the shaft of the femur, combined with hip dislocations, are extremely rare injuries.
• This article presents a case never described before of an unusual combination of an irreducible posterior hip fracture-dislocation associated with an ipsilateral femoral shaft fracture.
• Closed reduction attempts were unsuccessful, leading to the requirement for open reduction, which revealed that the femoral head buttonholed through the capsule.
• Timely diagnosis, careful assessment, and consideration of closed and open reduction techniques are essential in managing these complex injuries.

## 1 Introduction

Ipsilateral fractures of the shaft of the femur combined with hip dislocations are extremely rare injuries [1]. Currently, posterior hip fractures with ipsilateral femur fractures occur in only 1 in 100,000 [[2], [3], [4], [5]]. There is always a challenge in choosing the treatment to reduce hip and the right method of fixation for a femur fracture [4]. Initial diagnosis of posterior hip fracture is delayed in approximately half of all case because it focuses on overt hip fracture and resultant disability [1]. The emergency room and trauma team must carefully examine both injuries to properly identify them [4,5]. The reduction of the hip is very challenging in this situation due to the association of long bone fractures [6].

Only 3 % of hip dislocations are irreducible, according to Canale and Manugian's study [7]. Furthermore, the association with a femoral shaft fracture is extremely uncommon. We report a case of a femoral shaft fracture and ipsilateral irreductible posterior hip fracture – dislocation, where the femoral head crossed over a buttonhole through the capsule and did not cross the edge of the acetabulum, following a vehicular accident. To the best of our knowledge, this complex injury has not been previously reported in the literature.

## 2 Case presentation

The report writing and presentation of this study follow the SCARE 2023 criteria [8], ensuring adherence to guidelines for quality reporting in case report.

Informed consent was obtained from the patient for the use of his information, images, and intraoperative photos. All procedures performed in this study were in accordance with the ethical standards of the institutional research committee and with the Helsinki declaration [9] and its later amendments.

A 19-year-old patient with no previous medical history was admitted to emergency following a road traffic accident resulting in closed trauma to the left hip and thigh with pain and total functional disability.

**Fig. 1:**
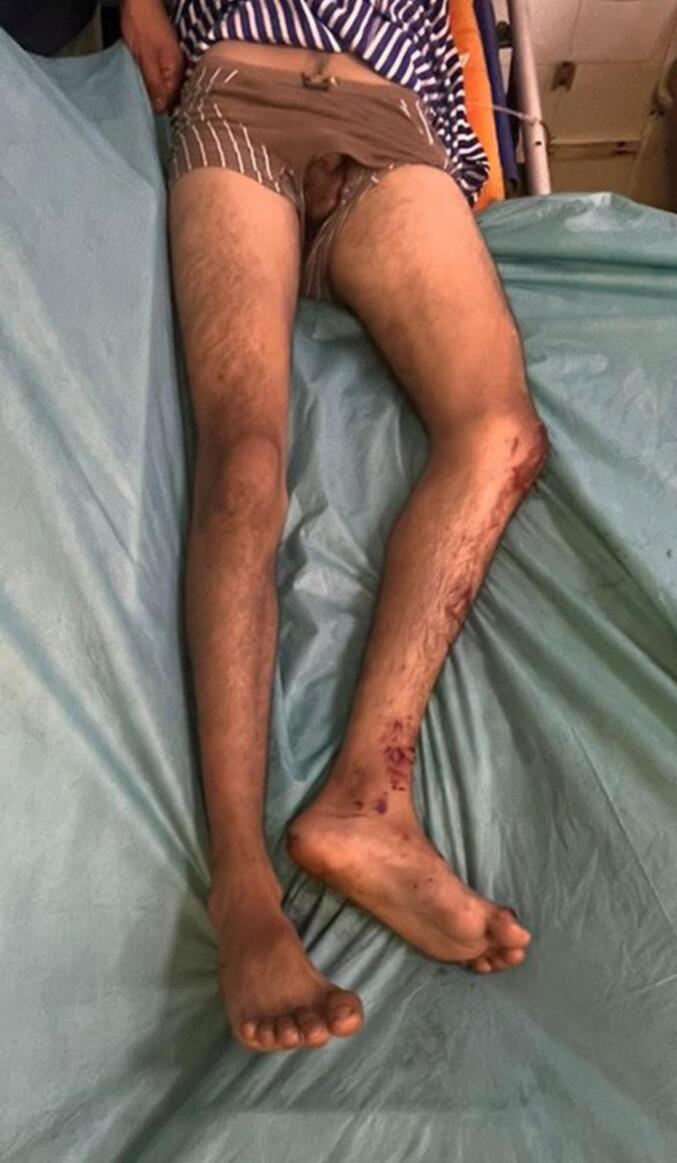
Picture of the patient's clinical attitude.

The patient described severe pain in his pelvic area as well as acute pain running down his leg. The patient reported extensive generalized pain in his pelvic region, as well as sharp pain down his thigh. The patient's vital signs remained stable at the time of admission, and he was able to answer questions. On clinical examination, we found that he had flexion-adduction deformity of the left hip (Fig. 1). His left foot had external rotation and abduction, and his left leg seemed to be slightly shorter than his right leg. There was no evidence of a distal neurovascular impairment.

**Fig. 2:**
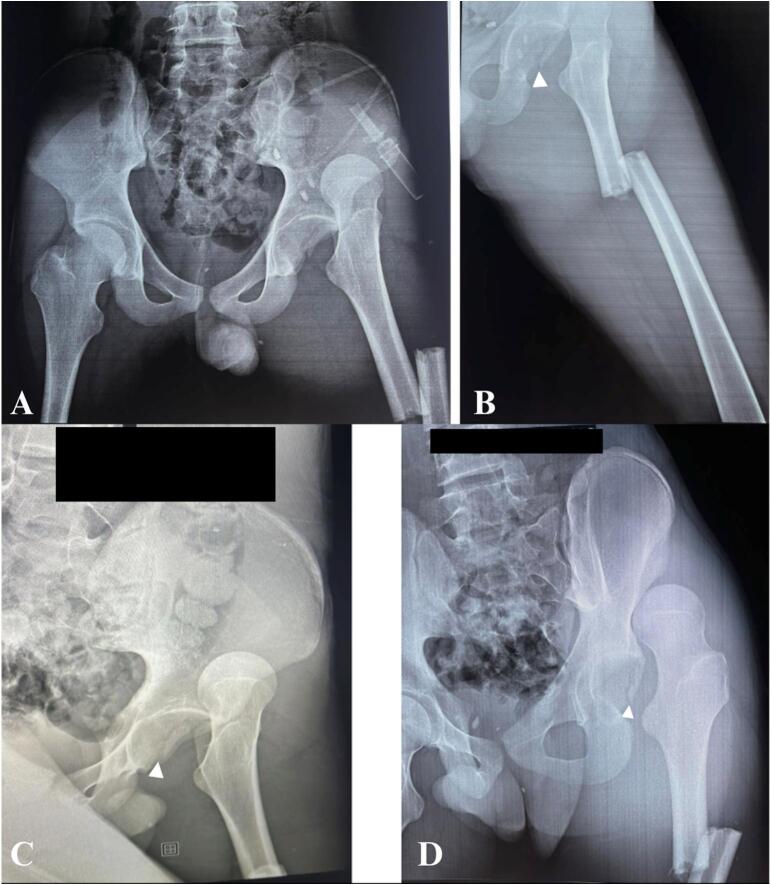
A + B + C + D: Radiographs demonstrating a left hip fracture dislocation with an acetabular posterior wall fracture (white arrows) associated with left femoral and shaft fractures (C: Iliac oblique view, D: Obturator oblique view).

After providing advanced trauma life support, the patient was sent for radiography. Radiographs were obtained of the pelvis, the left hip and femur, demonstrating a left hip fracture dislocation with an acetabular posterior wall fracture associated with left femoral and shaft fractures (Fig. 2). The CT scan was not performed at the time due to technical issues.

Within 3 h, the patient underwent, under general anesthesia, several attempts at closed reduction by external maneuvers combining traction in flexion, adduction, internal rotation, correction of rotation and adduction and restoration of straightness of the hip.

Despite these manoeuvers, the irreducibility of the hip required open reduction. During the same anesthesia the patient was positioned in supine position on X-ray transparent operating table.

We first inserted two 5 mm apex pins, fixed to a 4-hole pin clamp, into the subtrochanteric cortex percutaneously under fluoroscopic guidance. This was to manipulate the femoral head into the acetabulum using the joy-stick technique but were ultimately not successful.

**Fig. 3:**
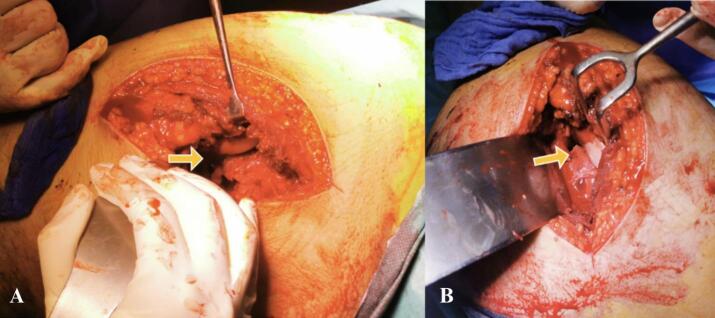
A + B: Shows a posterior dislocation of the femoral head buttonholed through the capsule with a capsular tear incarcerated in the acetabulum (arrow).

Hence, the patient was positioned in the right lateral decubitus; the left hip was approached via the posterolateral approach. This revealed posterior dislocation of the femoral head buttonholed through the capsule. There was an osteochondral fragment measuring 30 mmx10 mm attached to the labrum with a 3 cm capsular tear incarcerated in the acetabulum (Fig. 3). The piriformis muscle was ruptured 1 cm from its trochanteric insertion. After excision of the labrum and capsule, the reduction was successful. The labrum was sewn up to the soft tissues, the capsule was repaired using absorbable sutures and the osteochondral fragment was fixed with a 3.5 mm cortical screw.

The piriformis muscle was reinserted at the level of the greater trochanter, and the pelvitrochanteric muscles cut during the approach were sutured. Fluoroscan imaging confirmed the concentric reduction.

Subsequently, the femur shaft fracture was fixed with a closed reduction and an internal fixation with an intramedullary femur static interlocking nail (Auxein® Expert Femoral Nailing System; diameter 10 mm x Length 420 mm, catalog number: A3-REG-QF-13-F9).

**Fig. 4:**
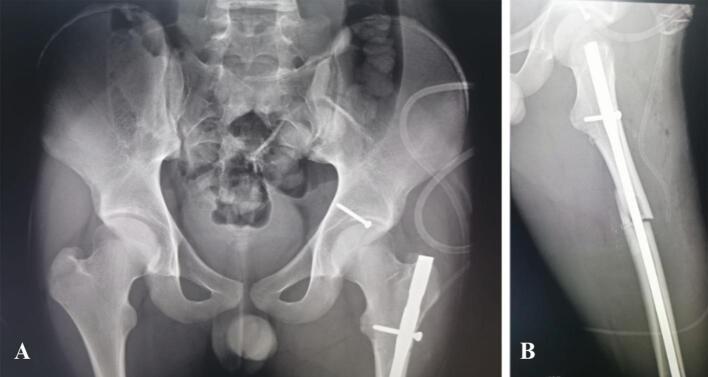
A + B: Post-reduction radiograph showing congruent reduction with a symmetrical widening of the joint space, with a reduction of the femoral shaft fracture.

The post op X-ray showed a symmetrical widening of the joint space, with a reduction of the femoral shaft fracture (Fig. 4).

The post-operative recommendations were for non-weight-bearing activity with axillary crutch walking for 6 weeks, followed by partial weight-bearing on fracture with union of femoral shaft.

**Fig. 5:**
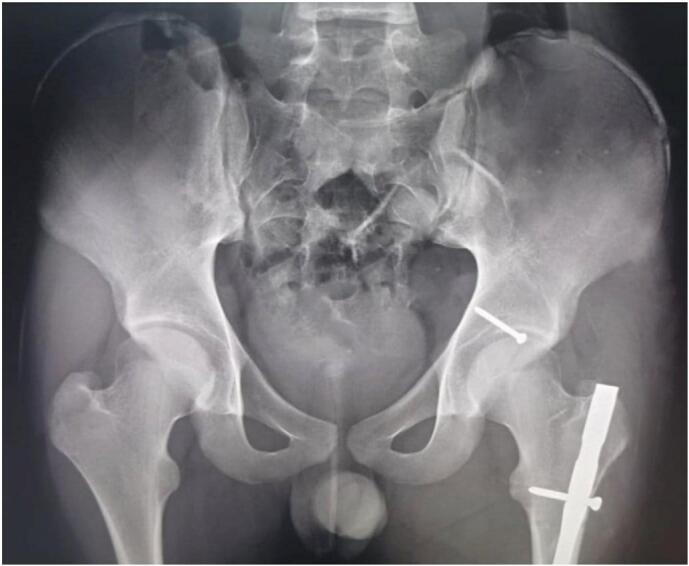
Radiographs at the last follow-up revealed no evidence of avascular necrosis of the femoral head.

After 19 months postoperative follow-up, the patient had full range motion of the affected hip without any pain. At the last follow-up the radiographs revealed no evidence of avascular necrosis of the femoral head (Fig. 5).

## 3 Discussion

Simultaneous head dislocation and ipsilateral femur shaft fracture are extremely rare [4]. High impact road traffic accidents have been reported to have caused these rare injuries [[2], [3], [4],^10^,^11^]. The association of an irreducible hip dislocation is even more rare and to the best of our knowledge has never been reported in the literature. There has been no consensus on how to treat such injuries [4]. The mechanism of the injury usually consists of hip dislocation and subsequent adduction result in a femur fracture. The injury mechanism is also presumed to be a horizontal compression on the flexible and adducted hip [11,12]. Hip dislocation is often missed to be diagnosed [13]. A proper clinical evaluation and radiological examination can help to avoid this [4]. The lesion may be different from pure dislocation to acetabular lip or femoral head fracture depending on the degree of hip adduction and flexion. For Paterson [14], however, as the flexion and adduction decreased, the size of the broken piece of the back wall increased [7].

Delay in hip dislocation often leads to delayed treatment, and open fractures may result in avascular necrosis of the hip [13,15]. Neurologic injury typically goes with traumatic dislocation, with the peroneal branch of the sciatic nerve injured in many posterior hip fracture dislocation [16].

Reduction of a hip dislocation is an emergency, but it is difficult to perform as it is not possible to use the fractured femur as a lever for the reduction of hip [4,11,12,17,18]. Furthermore, reduction failure may also be caused by fracture fragments or soft tissue in the acetabulum. CT and MRI examination of the acetabulum may be helpful in determining whether there are fracture fragments or soft tissue within the acetabulum if a failure to reduce hip dislocation occurs [18].

Compared to open reduction, closed reduction appears to have better clinical outcomes, possibly due to less disruption of the remaining blood supply to the femoral head [19].

Currently, there is limited literature on the treatment of these types of combination injuries. The surgical treatment will vary according to the skill and training of the surgeon. Presently, closed reduction is recommended over open reduction because it minimises disruption of the femoral head's remaining blood supply [20] and the complications frequently linked to open reduction [11,12,16].

Wiltberger appears to have described the first closed reduction technique in 1948 [2] for posterior hip dislocations associated with ipsilateral femoral shaft fractures. Another closed reduction technique was introduced in 1954 by Ingram et al. [21]. He inserted a large Steinmann pin anteroposteriorly through the greater trochanter, clamping it at the skin's border anteriorly and laterally with large vice-grip pliers. Strong manual traction was then used to achieve closed reduction. This effective technique has a high risk of damaging the sciatic nerve at the time of inserting the Steinmann pin percutaneously due to the greater trochanter's nonanatomic position. This method works well, however due to the greater trochanter's nonanatomic position, there is a significant danger of damaging the sciatic nerve while the greater trochanter is transfixed percutaneously. Two patients with hip dislocation were successfully treated in 1982 by Harper et al. [22], who placed the Steinmann pin in a posterior-anterior orientation while being careful to remain lateral to the sciatic nerve.

Closed reduction has been suggested by some surgeons, who manipulate the proximal fracture fragment using devices like the Scuderi traction screw [20], Smith traction screw [23], large bone clamp [24], Lardennois hoop [25], tourniquet [26], Hoffmann half pin [19], and temporary external fixator [27].

The Schanz screw has traditionally been the most used reduction tool [[28], [29], [30], [31], [32]]. It is attached to a universal AO chuck or T handle universal chuck and inserted percutaneously under fluoroscopic guidance in order to manipulate the proximal fracture fragment and contribute in reduction.

An original method for performing closed manipulation of a hip dislocation with a femoral shaft fracture was suggested by Rana et al. [4] and Iftekhar et al. [5]. Instead of managing the proximal fracture fragment, they temporarily fixed the femur with an external fixator in order to restore the femur's leverage.

Although numerous closure reduction approaches have been documented and have been shown to be effective, they are not always successful [33]. Open reduction should never be abandoned as an option, and its significance cannot be overstated as shown in many publications [1,18,34,35].

Several publications have reported the irreducibility of femoral head that occur after posterior dislocation [36,37].

The encasement of soft tissues of many origins can be attributed to the irreducibility of a pure dislocation: through the capsule buttonholed 4,10 the piriformis muscle wrapped around the femoral neck [38], acetabular obstruction caused by obturator internus, gemellus superior and inferior muscles [39].

Also, a large interposed intra-articular fragment from acetabular wall or the femoral head can lead to irreducible dislocations [[40], [41], [42]].

A CT scan is required to detect any osteochondral lesions, such as femoral head impaction, which may affect the hip's long-term functional prognosis [43]. Kim et al. [44], on the other hand, used an arthro-CT or MRI to identify the interposition of soft parts and/or capsule and labrum injury. In our case, open surgical reduction offered the best opportunity to visualize the acetabulum by decoaptation of the hip [7].

Forced closed reduction may aggravate these concurrent fractures or result in iatrogenic bone and peripheral neurovascular damage [45,46]. Several attempts at closed reduction are not suggested [47]. They may cause additional severe injury to the femoral head's chondral surface 3 and extend the risk of iatrogenic femoral neck fracture [48]. In this case, an open reduction should be undertaken as an option after a failed closed reduction attempt for hemodynamically stable patients [17]. If damaged tissues and fractures need to be repaired, the surgical reduction should be performed immediately via a posterior approach to the hip, rather than an anterior or anterolateral approach [7]. However, the vascular risk to the medial circumflex artery is significant, putting the patient at risk of secondary cephalic necrosis [49].

If possible, osteosynthesis of the small bone pieces adhering to the labrum and capsule should be restored. Several authors have effectively underlined the significant role of the capsule and labrum in the posterior stability of the hip [[50], [51], [52], [53]]. This explains why it is critical to fix these two aspects in a systematic way [49].

During the postoperative phase Early mobilization and weight-bearing may improve functional outcomes [7].

## 4 Conclusion

Ipsilateral femoral shaft fracture and hip dislocation are rare injuries that are always difficult to diagnose and treat, necessitating an exhaustive physical examination and radiographic imaging. These injuries are always regarded as orthopedic emergencies.

Attempts at closed reduction, using various external reduction devices to manage the proximal fracture fragment or immediately repair the femoral leverage, have been demonstrated to be effective in specific cases.

In cases of irreducible posterior dislocations, the surgical posterior approach must be used straight away to remove the interposition. Injury to the capsule, labrum, and muscles should be corrected.

## Consent

Written informed consent was obtained from the patient for publication of this case report and accompanying images. A copy of the written consent is available for review by the Editor-in-Chief of this journal on request.

## Provenance and peer review

Not commissioned, externally peer reviewed.

## Ethical approval

Informed consent for anonymized publication obtained from patient. Case report contributes to medical knowledge without additional procedures.

## Funding

This research did not receive any specific grants from funding agencies in the public, commercial, or not-for-profit sectors.

## Guarantor

The grantor for this case report is the first author and the corresponding author doctor Imad Jadib.

## CRediT authorship contribution statement

All the authors contributed to the study concept, data analysis, and writing of the paper.

## Declaration of competing interest

The authors report no declarations of interest.
